# Cranial autonomic symptoms in episodic and chronic migraine: a cross sectional study in Iran

**DOI:** 10.1186/s12883-021-02513-0

**Published:** 2021-12-20

**Authors:** Mansoureh Togha, Elham Jafari, Atieh Moosavian, Abolfazl Farbod, Shadi Ariyanfar, Fatemeh Farham

**Affiliations:** 1 Headache Department, Iranian Center of Neurological Research, Neuroscience Institute, Tehran University of Medical Sciences, Tehran, Iran; 2 Department of Clinical Nutrition and Dietetics, Faculty of Nutrition and Food Technology, Shahid Beheshti University of Medical Sciences, Tehran, Iran

**Keywords:** Migraine, Cranial autonomic symptoms, Blurred vision, Trigeminal autonomic cephalalgias

## Abstract

**Background:**

Cranial autonomic symptoms are common in migraine, with eye redness and tearing being the most common ones. Their identification can help to avoid misdiagnosis, predict the disease course, and select the appropriate treatment.

**Methods:**

This was a cross-sectional study of 904 patients who presented with migraine to a headache referral clinic. The participants filled out a questionnaire about their headache characteristics, as well as the presence of cranial autonomic symptoms. A total of 904 patients, 698 women (77.2%) and 206 men (22.8%), were included in the study, with a mean (SD) age of 38.05 (11.76) years.

**Results:**

About 70% of subjects with chronic migraine and 56.2% of those with episodic migraine reported one or more cranial autonomic symptoms. The two most commonly reported autonomic symptoms were eye redness (36.06%) and tearing (21.02%). Chronic migraine (43.4% vs. 29.5%), unilateral headache (56.8% vs. 48.7%), and blurred vision (20% vs. 14.7%) were significantly more frequent in migraineurs with cranial autonomic symptoms. Headache intensity and frequency in subjects with cranial autonomic symptoms were significantly higher than in those without cranial autonomic symptoms.

**Conclusion:**

We found higher percentages of cranial autonomic symptoms in patients with unilateral headaches, frequent and severe attacks and blurred vision. A diagnosis of cranial autonomic symptoms accompanying migraine may predict more severe disease and the possibility of evolution into chronic migraine.

## Introduction

Migraine is a common disabling disorder with a worldwide prevalence of 7 to 18% [1]. The primary characteristics of migraine are a unilateral and/or throbbing headache associated with nausea/vomiting +/− photophobia and phonophobia. Typical migraine attacks can be associated with cranial autonomic symptoms (CAS), which are the hallmark of trigeminal autonomic cephalalgias (TAC) [2]. The reported prevalence of CAS in migraine varies from 27 to 73% [3, 4]. This rate is even higher in patients with chronic migraine. CAS include eye redness, tearing, nasal congestion, rhinorrhea, eyelid edema, forehead and facial sweating, miosis, and ptosis [2].

Migraineurs experiencing CAS are more likely to experience unilateral headaches and more severe and frequent attacks [5, 6]. Prolonged peripheral sensitization in such patients appear to trigger the trigeminal autonomic reflex that contributes to the development of autonomic symptoms. Higher rates of photophobia, phonophobia, and osmophobia, as well as allodynia have been reported in migraineurs with CAS compared to those without autonomic features [4–9].

Unilateral autonomic symptoms can complicate the differentiation of unilateral migraine from TAC; however, CAS in migraine tend to be less severe and are usually bilateral and less consistent [9, 10]. Identification of autonomic symptoms in migraineurs in clinical practice is necessary to avoid misdiagnosis, predict the disease course, and select the appropriate treatment. This study aims to evaluate the frequency and characteristics of CAS in patients with episodic and chronic migraine and their relation to disease course.

## Methods

### Study population

In this cross-sectional study, 904 patients with migraine who presented to a headache referral clinic between April 2019 and September 2020 were included. All of the participants had reported at least five episodes of migraine within the last year. Episodic or chronic migraine was diagnosed by an expert neurologist/headache specialist according to the criteria of the International Classification of Headache Disorders-3 (ICHD3) [2]. Patients with medication overuse headache were also included.

### Data collection

After informed consent was obtained, the participants filled out a questionnaire about their headache characteristics. The patient demographic data, including age, sex, and disease duration, were recorded. Information was collected about headache-related features such as headache quality, frequency, intensity and duration. Headache intensity was scored by the patient using a numerical analog scale. The presence of aura and associated symptoms of nausea, vomiting, photophobia, phonophobia, and osmophobia also were specified. Visual phenomena that were not typical of migraine aura in terms of pattern and timing, were considered as blurred vision.

The cranial autonomic symptoms assessed in this study were eye redness, tearing, ptosis, lid edema, nasal congestion, and rhinorrhea as reported by the subject. Subjects who experienced one or more of these symptoms during a headache were considered to have CAS.

### Statistical analysis

The Stata software package V.14 for Windows was used for statistical analysis. Normal distribution of continuous measures was assessed using the Kolmogorov-Smirnov test and Q-Q plot. The student t-test or Mann-Whitney U test was used for continuous measures and the chi-square or Fisher’s exact test was used for categorical data. In this study, all *p*-values were two tailed and statistical significance was defined as *p* < 0.05.

## Results

A total of 904 patients, 698 women (77.2%) and 206 men (22.8%), were included in the study. The mean (SD) age of the participants was 38.05 (11.76) years and the average (SD) duration of illness was 14.22 (9.57) years. Eighty-seven percent of the subjects experienced migraine without aura. The subjects experienced an average (SD) of 11.87 (8.29) attacks per month. Their headaches were reported as bilateral by 46.3% of subjects and as unilateral by 53.7% of subjects.

**Fig. 1:**
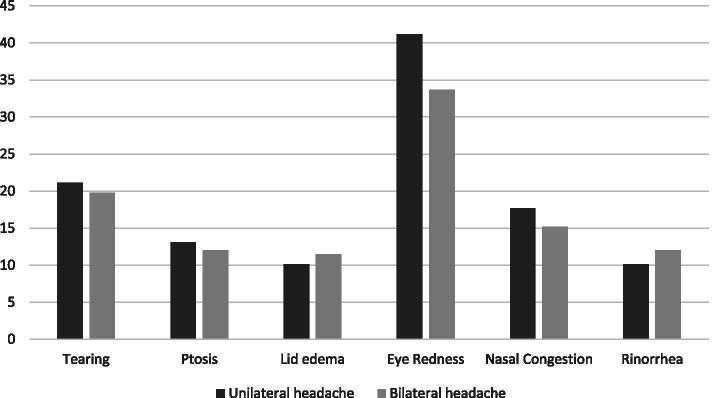
Percentage of autonomic symptoms according to migraine lateralization state. Prevalence differences between groups for all symptoms were not statistically significant (*p* > 0.05) except for eye redness (*p* = 0.03)

The overall prevalence (95% CI) of one or more CAS was 60.95% (57.68 to 64.15%). Seventy percent of subjects with chronic migraine and 56.2% of those with episodic migraine reported at least one CAS. The two most common autonomic symptoms experienced were eye redness (36.06%) and tearing (21.02%). The prevalence (95% CI) of ptosis was 13.16% (11.03 to 15.54%), of lid edema was 10.17% (8.28 to 12.33%), of nasal congestion was 15.93% (13.6 to 18.48%), and of rhinorrhea was 10.73% (8.79 to 12.93%). Figure 1 shows that the prevalence of eye redness in subjects with unilateral migraine was higher than in subjects with bilateral migraine. This difference was statistically significant (*p* = 0.03).

Table 1 lists the differences between subjects with and without CAS based on the headache characteristics. The proportions of chronic migraine (43.4% vs. 29.5%), unilateral headache (56.8% vs. 48.7%), and blurred vision (20% vs. 14.7%) in migraineurs with CAS were higher than in those without CAS and these differences were statistically significant (*p* < 0.05). Headache intensity and headache frequency in migraineurs with CAS were higher than in those without CAS and these differences were statistically significant (p < 0.05). Among patients with CAS, headache intensity and frequency were significantly higher in those who had more than one CAS (*P* < 0.001) (Table 2).

**Table 1:** Demographics and characteristics^a^ of subjects with and without cranial autonomic symptoms

Demographics and characteristics		CAS		*P*-value
		Absent (***n*** = 353)	Present (***n*** = 551)	
Age by year		37.14 (11.77)	38.62 (11.73)	0.07
Sex	male	80 (22.7)	126 (22.9)	0.94
	female	273 (77.3)	425 (77.1)	
Migraine subtype	episodic	222 (70.5)	286 (56.6)	**< 0.001**
	chronic	93 (29.5)	219 (43.4)	
Lateralization	unilateral	149 (48.7)	285 (56.8)	**0.03**
	bilateral	157 (51.3)	217 (43.2)	
Aura	no	258 (89.3)	404 (85.6)	0.14
	yes	31 (10.7)	68 (14.4)	
Nausea		199 (56.4)	324 (58.9)	0.45
Vomiting		78 (22.1)	137 (24.9)	0.33
Constipation		48 (13.6)	80 (14.5)	0.69
Phonophobia		238 (67.4)	386 (70.1)	0.4
Photophobia		243 (68.8)	390 (70.8)	0.53
Osmophobia		185 (52.4)	301 (54.6)	0.51
Blurred vision		52 (14.7)	110 (20)	**0.05**
Disease duration by year		13.43 (9.3)	14.7 (9.71)	0.07
Headache intensity		7.57 (1.86)	7.99 (1.76)	**0.001**
Headache frequency/month		10.29 (7.82)	12.84 (8.43)	**< 0.001**

^a^ Presented as number (valid percent) and mean (SD)

**Table 2:** Distribution of headache intensity and frequency, according to the number of cranial autonomic symptoms

Characteristic	CAS			*P* value
	No cas	1	> 1	
Headache intensity, median (IQR)	8 (6–9)	8 (6–9)	8 (7–10)	< 0.001
Headache frequency, median (IQR)	8 (4–15)	10 (5–15.25)	12 (6–20)	< 0.001

The frequencies of unilateral and bilateral CAS were determined in 568 patients. Among these, most symptoms were unilateral, except for eyelid edema and eye redness, which occurred bilaterally in a larger number of patients (Table 3).

**Table 3:** Prevalence^a^ of unilateral and bilateral cranial autonomic symptoms in 568 migraineurs

Characteristic	With CAS		Without CAS
	Unilateral symptom	Bilateral symptom	
Tearing	90 (15.8)	61 (10.7)	417 (73.4)
Ptosis	78 (13.7)	49 (8.6)	441 (77.6)
Lid edema	15 (2.6)	29 (5.1)	523 (92.2)
Eye redness	75 (13.2)	89 (15.7)	404 (71.7)
Nasal congestion	69 (12.1)	40 (7)	459 (80.8)
Rhinorrhea	45 (7.9)	30 (5.3)	493 (86.8)

^a^Presented as number (valid percent)

## Discussion

Cranial autonomic symptoms are considered to be diagnostic criteria for TAC; however, they have been reported in 27 to 73% of subjects with typical migraine [3–5, 9]. A recent study in Asia reported CAS in 42.4% of migraineurs [7]. The prevalence of CAS in the subjects in our study was 60.95%, with eye redness and tearing being the most common. Eye redness and tearing have been reported to be the most commonly detected CAS in other studies as well [4, 8, 11].

More than half of migraineurs with CAS report bilateral autonomic symptoms, especially those with bilateral headache [7]. Eye redness which was the most common CAS in our study, was more frequently bilateral, however, other autonomic symptoms were mostly reported unilaterally, except for eyelid edema. The unilaterality of headache has been reported to be an independent risk factor in migraineurs with autonomic symptoms [3, 4, 8]*.* We also found a significantly higher proportion of unilateral headache in our subjects with CAS than in those without CAS. Furthermore, the prevalence of eye redness in our subjects with unilateral migraine was significantly higher compared to those with bilateral headaches. Another study has indicated that the odds ratio (OR) of bilateral CAS for migraine versus cluster headache (CH) was highest for eye redness, followed by eyelid edema [9]. It can be inferred that bilateral eye redness is more common in migraine than in CH and that redness of the eyes occurs more commonly in unilateral migraine.

Emergence of unilateral autonomic symptoms can complicate the differentiation of unilateral migraine from TAC; however, CAS in migraine tend to be less severe, unrelated to the headache side, and less consistent. It should also be noted that only a small percentage of patients with CH report bilateral symptoms, except for facial sweating [9, 10].

The subjects in our study experienced an average of 11 migraine attacks per month. This high frequency of headaches can be attributed to the referential nature of our headache clinic. However, headache intensity and frequency were significantly higher in subjects with CAS and chronic migraine was diagnosed in a significantly higher number of these subjects. Similar findings about higher frequencies, longer attack durations, and more severe headache attacks in migraineurs with CAS have been reported in other studies; although there are few reports about the percentages of chronic migraine in such patients in the literature [3–6, 9, 12].

Among our patients with chronic migraine, 70% had experienced one or more CAS. This finding is similar to a study that found at least one CAS in 82% of patients with chronic migraine [11]. A severe intensity and the long duration of pain attacks in migraineurs with CAS could be the clinical consequence of an intense peripheral input to highly sensitized trigeminal neurons. There should be a certain threshold of pain after which the trigeminal system initiates parasympathetic activation and the appearance of CAS. This could be the reason why CAS are more prevalent in subjects with long-lasting illness and prolonged and severe attacks [4, 6, 12]. Furthermore, the severity of autonomic symptoms is also related to the intensity of pain, as we found a significant correlation between the number of autonomic symptoms, and intensity or frequency of headaches.

Migraineurs can experience a variety of negative or positive visual phenomena, as well as perceptual abnormalities which are not typical of a visual aura [13]. Blurred vision is the most frequently reported visual manifestation of migraine, with a frequency of 27 to 54.1% in different studies. It has been proposed that blurred vision could be a trigeminal autonomic symptom which arises from an imbalance between sympathetic and parasympathetic inputs [14]. We found blurred vision in 17.9% of subjects, with a significantly higher proportion in those with CAS than in those without CAS. The results of our study support the hypothesis that visual disturbances other than aura in migraineurs could be an autonomic symptom. However, previous studies on autonomic symptoms in migraine have not addressed this issue specifically and there is a need for further studies in this area.

Other associated symptoms that have been reported to have a significant relation to CAS include photophobia, phonophobia, osmophobia, nausea and allodynia [4, 6, 7, 9]. We found no significant association of CAS with any of these symptoms in the present study.

A new scale has been designed to evaluate and quantify CAS in primary headaches that can be included as a secondary efficacy endpoint when evaluating the response to treatment, but it has not been validated yet [15].

There is limited research on specific treatments for migraine accompanied by CAS, although, triptans, lidocaine, and onabotulinumtoxinA have demonstrated greater effects in this group compared to other migraineurs. Triptans can improve autonomic symptoms such as lacrimation, eye redness, eyelid edema, nasal congestion, and rhinorrhea in addition to severe pain. The presence of CAS might also help predict the response to onabotulinumtoxinA, suggesting that it could reduce autonomic outflow [5, 16, 17].

Another issue that requires further research is the choice of prophylactic drug in migraineurs with autonomic symptoms. It remains to be assessed whether migraineurs with CAS have a better response to the drugs that are effective for TAC. A final point to be considered is the possible existence of autonomic symptoms in the diagnosis of migraine and how to differentiate them from the autonomic symptoms of TAC.

The strengths of the current study are the large sample size at a tertiary headache center, diagnosis of headache by a headache specialist or trained neurologist according to ICHD3 criteria, assessment of visual symptoms other than aura, and the inclusion of patients with chronic migraine in the evaluation of CAS. It is limited, however, by: (1) selection bias toward patients with a greater disease burden because of the specialized nature of the headache clinic; (2) self-reporting by subjects of autonomic symptoms, which could be imprecise; (3) recall bias, as most of the data were collected interictally; and (4) the possible influence of acute and preventive treatments on CAS.

## Conclusion

Cranial autonomic symptoms are common in migraine. We found higher proportions of CAS in unilateral headaches and in patients with frequent and severe attacks. Blurred vision was more common in migraineurs with CAS, which may suggest it as an autonomic symptom. The emergence of autonomic symptoms may be a warning sign for the progression to chronicity and the need for more serious prophylactic treatment. Further studies are needed about the treatment options for autonomic symptoms associated with headache.

## Data Availability

All data of this research is available upon reasonable request.

## Abbreviations

CAS: Cranial autonomic symptoms
TAC: Trigeminal autonomic cephalalgias
ICHD3: International Classification of Headache Disorders-3
TUMS: Tehran University of Medical Sciences
CH: Cluster headache
OR: Odds ratio
